# An analog sensitive allele permits rapid and reversible chemical inhibition of PKC-3 activity in *C. elegans*

**DOI:** 10.17912/micropub.biology.000610

**Published:** 2022-08-04

**Authors:** KangBo Ng, Tom Bland, Nisha Hirani, Nathan W. Goehring

**Affiliations:** 1 Francis Crick Institute, London, NW1 1AT, UK; 2 Institute for the Physics of Living Systems, University College London, WC1E 6BT, UK

## Abstract

Engineered analog sensitive kinases provide a highly effective method for acute, controllable, and highly selective inhibition of kinase activity. Here we describe the design and characterization of an analog sensitive allele of the polarity kinase, PKC-3. This allele supports normal function as measured by its ability to exclude PAR-2 from the anterior membrane of zygotes, and is rapidly and reversibly inhibited in a dose-dependent manner by the ATP analog 1NA-PP1. This allele provides a new tool to explore the role of PKC-3 in diverse contexts within *C. elegans* , particularly those in which acute and reversible control of PKC-3 kinase activity may be desired.

**Figure 1. Design and characterisation of an analog sensitive allele of C. elegans pkc-3:**
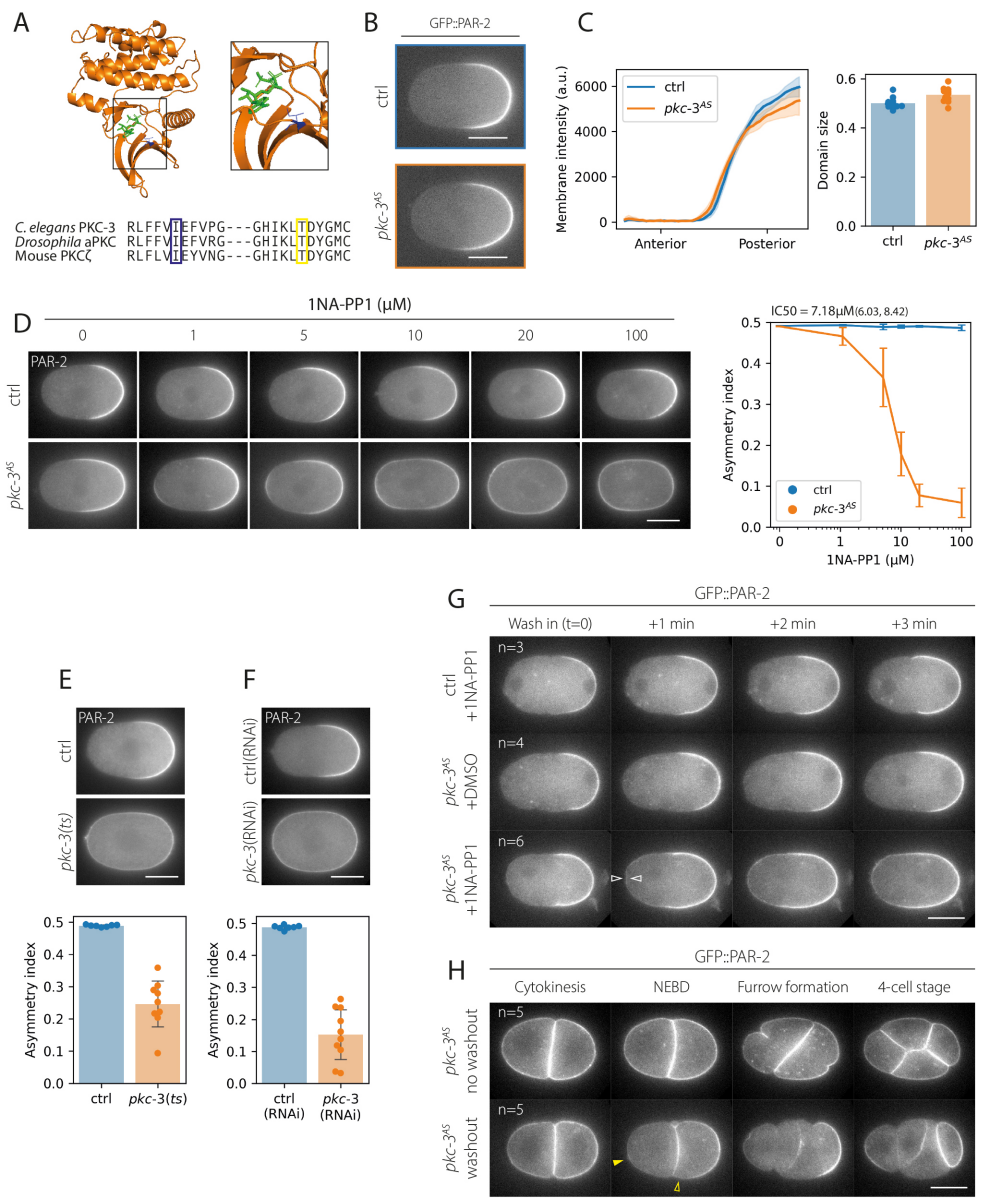
**(A)** **Structure and homology of the PKC-3 active site.** Predicted structure of the *C. elegans* PKC-3 kinase domain (AlphaFold) showing gatekeeper residue (blue). Superposition of the bound ATP analogue (green) obtained by structural superposition with PKC beta (pdb code 3PFQ) as in Hannaford et al. (2019). Amino acids around the position of the gatekeeper site (blue) and the suppressor site (yellow) appear well conserved. **(B, C) The analog sensitive PKC-3, PKC-3 ^AS^ , excludes PAR-2 from the anterior as efficiently as wild-type.** Example midsection confocal images shown along with quantification of PAR-2 membrane concentration profiles and PAR-2 domain size for *pkc-3 ^AS^* (n=11) and wild-type control embryos (n=12). **(D) Inhibition of PKC-3 ^AS^ by the ATP analogue 1NA-PP1 results in dose-dependent loss of PAR-2 asymmetry.** Example midsection confocal images of control or *pkc-3 ^AS^* embryos expressing GFP::PAR-2 treated with the indicated concentration of 1NA-PP1. Quantitation of asymmetry index shown at right. Sample sizes: control - 0 uM (n=6), 1 uM (n=6), 5 uM (n=5), 10 uM (n=4), 20 uM (n=5), 100 uM (n=5); *pkc-3 ^AS^ -* 0 uM (n=5), 1 uM (n=5), 5 uM (n=6), 10 uM (n=6), 20 uM (n=6), 100 uM (n=5). IC50 was calculated by bootstrapping to obtain a mean and a 95% confidence interval, mean = 7.18, bounds = 6.03, 8.42. **(E, F) Inhibition of PKC-3 ^AS^ is as efficient or better at reducing PAR-2 asymmetry compared to established methods.** For comparison, asymmetry index of PAR-2 is shown for the temperature sensitive *pkc-3(ne4246)* allele at the restrictive temperature (control, n=7; *pkc-3(ts)* , n=9) (E) and after RNAi targeting *pkc-3* (control(RNAi), n=7; *pkc-3* (RNAi), n=10) (F). **(G) Inhibition of PKC-3 ^AS^ is rapid.** A timeseries of midsection confocal images of either control or *pkc-3 ^AS^* embryos expressing GFP::PAR-2 treated with 1NA-PP1 or DMSO (control). Open arrowheads (white) indicate ectopic GFP::PAR-2 localisation at the anterior pole, which begins to appear by one minute after wash-in of 1NA-PP1. **(H) Inhibition of PKC-3 ^AS^ is reversible.** GFP::PAR-2-expressing embryos were treated with 1NA-PP1 (50uM) at the one-cell stage to prevent polarization. Embryos were allowed to divide symmetrically and then subject to buffer exchange in the presence or absence of 1NA-PP1 immediately following cytokinesis. Reactivation is evident by the formation of a polarized PAR-2 cap (closed arrowhead, yellow) and clearance of PAR-2 in the anterior (open arrowhead, yellow). Scale bars, 20μm. Individual data points or mean ± SD shown for all plots.

## Description

Much of our understanding of PAR polarity comes from experiments performed in *C. elegans* zygotes, in which RNAi allows highly efficient depletion of the relevant players, including the key polarity kinase atypical protein kinase C (PKC-3 in *C. elegans* ). Indeed, work in the zygote has revealed the basic paradigm by which PKC-3 restricts posterior PAR proteins (pPARs) from invading the anterior domain of the zygote through phosphorylation-dependent dissociation of PAR-1, PAR-2 and LGL-1 from the plasma membrane (Lang and Munro, 2017). Until relatively recently, analysis of PKC-3 function beyond the zygote was hampered by the lack of methods to reliably deplete or inhibit PKC-3 function at specific times in development. This has changed with the identification of small molecule inhibitors, temperature sensitive alleles, and advances in tissue specific and auxin-inducible degradation systems (Rodriguez *et al.* , 2017; Castiglioni *et al.* , 2020; Fievet *et al.* , 2013; Montoyo-Rosario *et al.* , 2020). Small molecule inhibitors of PKC-3 hold particular promise as they allow a unique combination of rapid, specific, and reversible suppression of PKC-3 function. However, the only PKC-3 inhibitor demonstrated to work efficiently in *C. elegans* , CRT0103390 (Rodriguez *et al.* , 2017), is proprietary, has been difficult to obtain, and is unfortunately no longer being made available by Cancer Research UK.

To address this unmet need for reliable inhibitors of PKC-3 activity, we pursued a chemical-genetics approach based on engineering the ATP binding pocket to be selectively sensitive towards bulky non-hydrolysable ATP analogues (Bishop *et al.* , 2000). This approach relies on modification of a key gatekeeper residue that gates access to the enzyme active site and has been successfully used to create analog sensitive variants of aPKC in a variety of systems, including zebrafish, mouse and *Drosophila* (Cibrián Uhalte *et al.* , 2012; Kumar *et al.* , 2015; Hannaford *et al.* , 2019).

To generate a *pkc-3* analogue-sensitive (as) allele in *C. elegans* , we identified the putative gatekeeper site as isoleucine 331 based on the *Drosophila* aPKC ^as4^ , structural modeling and sequence homology ( **Fig. 1A** ). We noted that in *Drosophila* aPKC ^as4^ , mutation of the predicted gatekeeper site resulted in lethality, suggesting that this mutation compromised kinase activity. Lethality was rescued by additionally mutating the amino acid immediately prior to the DFG motif (the so-called suppressor site) to an alanine, which has been previously shown to improve kinase function in the context of gatekeeper mutations (Blethrow *et al.* , 2004; Hannaford *et al.* , 2019). In an identical approach, we used CRISPR to generate a *pkc-3* allele that contained both mutations: I331A (gatekeeper site) and T394A (suppressor site), and obtained homozygous viable worms ( **Fig. 1A** ). Hereafter we refer to PKC-3[I331A; T394A] as PKC-3 ^AS^ .

To confirm both the function and our ability to inhibit PKC-3 ^AS^ , we used localization of posterior PAR protein PAR-2 in zygotes as a readout. PAR-2 is a known substrate of PKC-3 and its exclusion from the anterior membrane of zygotes is a sensitive readout of PKC-3 activity. To confirm the function of PKC-3 ^AS^ , we therefore quantified PAR-2 localisation during the polarity maintenance phase in *pkc-3 ^AS^* and control *pkc-3(wt)* zygotes. Results were largely indistinguishable, suggesting that *pkc-3 ^AS^* embryos possess normal PKC-3 activity ( **Fig. 1B, C** ).

We next measured the response of PKC-3 ^AS^ to the ATP analogue 1NA-PP1 *in vivo* by treating *pkc-3 ^AS^* and *pkc-3(wt)* zygotes expressing GFP::PAR-2 with increasing concentrations from 0-100 µM ( **Fig. 1D** ). Plots of PAR-2 asymmetry in *pkc-3 ^AS^* zygotes as a function of 1NA-PP1 revealed a clear concentration-dependent loss of asymmetry, yielding an effective IC50 of approximately 7.18 µM. Control zygotes showed effectively no change over the full range of tested 1NA-PP1 concentrations. To benchmark PKC-3 inhibition with respect to existing methods, we quantified GFP::PAR-2 asymmetry in *pkc-3(ts)* at the restrictive temperature and *pkc-3(RNAi)* zygotes. At least with respect to GFP::PAR-2, these data suggest that these conditions roughly equate to 10-20 µM 1NA-PP1 ( **Fig. 1D-F** ).

Finally, we used acute wash in/wash out experiments to assess the timescale and reversibility of PKC-3 ^AS^ inhibition by 1NA-PP1. To investigate the response time of PKC-3 ^AS^ to 1NA-PP1 inhibition, we washed in i) 1NA-PP1 into *pkc-3(wt)* embryos (drug control), ii) DMSO into *pkc-3 ^AS^* embryos (allele control) and iii) 1NA-PP1 into *pkc-3 ^AS^* embryos (investigation). We found that ectopic localization of GFP::PAR-2 was detectable at the anterior pole within one minute after addition of 1NA-PP1. Neither drug nor allele controls exhibited any anterior localization of PAR-2 ( **Fig. 1G** ). To test reversibility of 1NA-PP1 inhibition of PKC-3 ^AS^ , we treated zygotes to block polarization and allowed them to divide yielding symmetric two-cell stage embryos with GFP::PAR-2 uniformly localized to the cortex of both blastomeres. We then performed a buffer exchange in the presence or absence of 1NA-PP1. When 1NA-PP1 was washed out, we typically observed subsequent polarization of PAR-2 in at least one blastomere and PAR-2 was inherited asymmetrically in the following division ( **Fig. 1H** ). When 1NA-PP1 was kept in the medium, PAR-2 remained uniform and remained localized uniformly to the membrane in all four blastomeres following cell division.

In conclusion, we report the generation and functional characterization of an analogue-sensitive PKC-3 in *C. elegans* . We envisage that this tool will provide a highly effective addition to our toolkit for manipulating PAR polarity, particularly when the induction of acute and rapid effects is desirable.

## Methods

*Strains*

*C. elegans* strains were maintained on OP50 bacterial lawns seeded on nematode growth media (NGM) at 20˚C or 15˚C (for experiments involving *pkc-3(ts)* , **Fig. 1E** ) under standard laboratory conditions (Stiernagle, 1999). OP50 bacteria were obtained from CGC. Zygotes were obtained from hermaphrodites unless otherwise noted. Analysis of embryos precludes determination of animal sex. Strains used indicated below.

**Table:** 

Strain	Genotype	Source
KK1273	*par-2(it328[gfp::par-2]) III*	CGC/Ken Kemphues
NWG0316	*pkc-3(crk77[I331A,T394A]) II* ; *par-2(it328[gfp::par-2]) III*	This paper
NWG0124	*pkc-3(ne4246) II* ; *par-2(it328[gfp::par-2]) III*	This paper

*Strain construction*

Mutation by CRISPR-Cas9 was performed based on the protocol published by (Arribere *et al.* , 2014). Briefly, tracrRNA (IDT DNA, 0.5 µL at 100 µM) and crRNA(s) for the target (IDT DNA, 2.7 µL at 100µM) with duplex buffer (IDT DNA, 2.8µL) were annealed together (5 min, 95˚C) and then stored at room temperature until required. An injection mix containing Cas9 (IDT DNA, 0.5µL at 10mg/mL), annealed crRNA, tracrRNA, and the repair template (IDT Ultramer) was incubated at 37˚C for 15 min and centrifuged to remove debris (10 min, 13,000 rpm) (see below). Young gravid KK1273 adults were injected along with a *dpy-10* or *unc-58* co-CRISPR injection marker (Arribere et al., 2014) and mutants identified by PCR and sequence verified.

**Table:** 

Oligos for *pkc-3* (T394A) suppressor site	
*pkc-3* (T394A) crRNA #1	AATGTTCTGATTGACGCTGA AGG
*pkc-3* (T394A) crRNA #2	CACATAAAACTGACAGATTA TGG
*pkc-3* (T394A) repair template (AluI restriction site introduced)	TCATTCTCGCGGAATCATTTATCGTGATCTCAAATTGGAC AACGTCTTAATCGATGCAGAAGGACATATCA **AGCT** AGCA GACTACGGAATGTGTAAAGAGAATATTAAGGATGGAGA TTTAACTTCA
Oligos for *pkc-3* (I331A) gatekeeper site	
*pkc-3* (I331A) crRNA #1	GCTTCCGTTGCTGTTGCATG TGG
*pkc-3* (I331A) crRNA #2	CGAATTCGATGACAAAGAAC AGG
*pkc-3* (I331A) repair template (MspI restriction site introduced)	AAAATTTTAAACCAAAATTTTGATAATTTCAGACCGAATC **CCGG** CTGTTCTTTGTCGCAGAATTCGTTCCTGGAGGTGAT CTGATGTTTCACATGCAACAGCAACGGAAGCTTCCAGAA GAGCACGCTCGA
Primers for genotyping (covers both I331A and T394A sites)	
Forward primer (screening)	CCACAATTCCCTTCCTCGCT
Reverse primer (screening)	TCGAATGGAGATCGACCAGC
* underlined base pairs in the crRNA denotes the PAM sequence, which needs to be removed before purchasing crRNA from IDT * **Bolded base pair** denotes where the restriction site was introduced.	

*Experiments – RNAi culture conditions*

RNAi by feeding was performed according to previously described methods (Kamath and Ahringer, 2003). Briefly, HT115(DE3) bacterial feeding clones were inoculated from LB agar plates to LB liquid cultures and grown overnight at 37ºC in the presence of 50 μg/mL ampicillin (until a fairly turbid culture is obtained). To induce high dsRNA expression, bacterial cultures were then treated with 1 mM IPTG before spotting 150μL of culture onto 60 mm NGM agar plates (supplemented with 10 μg/ml carbenicillin, 1 mM IPTG) and incubated for 24 hr at 20ºC. To permeabilize embryos for drug treatment, L3/L4 larvae were added to *ptr-2* RNAi feeding plates and incubated for 24-32 hours at either i) 20ºC for dose-response experiments ( **Fig. 1 D** ) or ii) 25ºC for acute inhibition/washout experiments ( **Fig. 1G, H** ) (Carvalho *et al.* , 2011). For knockdown of *pkc-3* , L3/L4 larvae were added to *pkc-3* RNAi feeding plates and incubated for 24-32 hours at 25ºC ( **Fig. 1F** ).

*Experiments – dissection and mounting for microscopy*

Embryos were dissected in 8-10 µL of Shelton’s Growth Medium (for permeabilized embryos) (Edgar and Goldstein, 2012) or egg buffer (118 mM NaCl, 48 mM KCl, 2 mM CaCl _2_ 2 mM MgCl2, 25 mM HEPES, pH 7.3; for temperature sensitive/ RNAi experiments), and mounted with 20 µm polystyrene beads (Polysciences, Inc.) between a slide and coverslip as in (Rodriguez *et al.* , 2017), and sealed using VALAP (1:1:1, vaseline:lanolin:paraffin wax).

*Experiments – drug treatment*

For the dose response experiments ( **Fig. 1D** ), embryos were dissected in Shelton’s Growth Medium containing 1NA-PP1 (Calbiochem, 529579). Embryos that had not yet completed meiosis II were tracked and imaged during maintenance phase.

For acute drug treatment, embryos were dissected as above but using 18.8 µm polystyrene beads instead, and mounted between a large and small coverslip sealed on two parallel edges with VALAP as in (Goehring *et al.* , 2011). Drug was introduced to the sample by capillary action by placing a drop of drug-containing solution at one side of the sample, and touching a piece of filter paper at the opposite side. For **Fig. 1G** and **H** , 50uM of 1NA-PP1 was used.

*Experiments ­– temperature upshift for pkc-3(ts) alleles*

For experiments involving the *pkc-3(ts)* allele ( **Fig. 1E** ), adult worms were transferred to a 27ºC incubator 3-6 hours before imaging.

*Microscopy*

Midsection confocal images were captured on a Nikon TiE with a 100x/1.40 NA oil objective, further equipped with a custom X-Light V1 spinning disk system (CrestOptics, Rome, Italy) with 50 μm slits, Obis 488/561 fiber-coupled diode lasers (Coherent, Santa Clara, CA) and an Evolve Delta EMCCD camera (Photometrics, Tuscon, AZ). Imaging systems were run using Metamorph (Molecular Devices, San Jose, CA) and configured by Cairn Research (Kent, UK). Filter sets were from Chroma (Bellows Falls, VT): ZT488/561rpc, ZET405/488/561/640X, ET535/50m, ET630/75m. All embryos were imaged with a 20ºC temperature collar, except for the temperature sensitive experiments, to which the temperature collar was set at 25.5ºC.

*Image analysis*

Raw or SAIBR processed images were used for quantification (Rodrigues *et al.* , 2022). In order to measure cortical concentrations, a 100-pixel-wide (15.5 μm) line following the membrane around the embryo was computationally straightened, and a 20-pixel-wide (3.1 μm) rolling average filter was applied to the straightened image. Intensity profiles perpendicular to the membrane at each position were fit to the sum of a Gaussian component, representing membrane signal, and an error function component, representing cytoplasmic signal, and a constant, representing background signal. Membrane concentrations at each position were calculated as the amplitude of the Gaussian component. This protocol is similar to previously published methods (Gross *et al.* , 2019; Reich *et al.* , 2019).

For calculating domain size, fluorescence profiles were first fitted to an error function with a defined gradient center and width. The domain size was calculated as *total length of fluorescence profile* – ( *gradient center* – 0.75 * *gradient width* ). Domain size is then defined as a fraction of the embryo perimeter.

For calculating asymmetry index, the following equation was used: Asymmetry index = (A – P) / (2 * (A + P)), where A and P denote the sum of fluorescence signals in the anterior or posterior 30% of the cell, respectively.

*Calculating IC50 for 1NA-PP1 dose response experiments*

To calculate IC50 ( **Fig. 1D** ), we randomly sampled data 10,000 times with replacement, and for each instance we obtained a mean, and fitted a 4 parameter logistic equation. The mean IC50 of the random samples was taken to be the approximated IC50, and confidence interval can be calculated from the spread of IC50 values.
